# Ultrasound-Assisted Extraction of Phenolic Compounds from Mango (*Mangifera indica* cv. Chok Anan) Peel and Its Inhibitory Effect on Enzymatic Browning of Potato Puree

**DOI:** 10.17113/ftb.57.03.19.5728

**Published:** 2019-09

**Authors:** Chotika Jirasuteeruk, Chockchai Theerakulkait

**Affiliations:** Department of Food Science and Technology, Faculty of Agro-Industry, Kasetsart University, 50 Ngam Wong Wan Rd, Lat Yao, Chatuchak, 10900 Bangkok, Thailand

**Keywords:** mango peel, polyphenol oxidase, potato, browning inhibition

## Abstract

Enzymatic browning is a serious quality deterioration of fresh-cut fruits and vegetables. Recently, consumers and fruit and vegetable industrial processors have demanded the use of natural antibrowning agents to replace the use of chemicals. Mango (*Mangifera indica* cv. Chok Anan) peel was prepared in the form of mango peel liquid nitrogen powder. This included extraction by ultrasound or ultrasound combined with stirring. The total phenolic content of mango peel liquid nitrogen powder extract (further in the text: mango peel extract) was the highest after the extraction for 15 min using ultrasound followed by stirring for 15 min. The browning value of potato puree treated with mango peel extract was lower, while its L* value and the hue angle were larger than of samples treated with ascorbic or citric acids during storage for 6 h. Mango peel extract had a competitive inhibitory effect on potato polyphenol oxidase (PPO), which was larger than either ascorbic or citric acid. Its IC_50_ value was 0.3 mg/mL. Mangiferin, protocatechuic and gallic acid found in mango peel extract had high inhibitory effect on potato PPO, making mango peel a potential natural source of enzymatic browning inhibitor.

## INTRODUCTION

Mango (*Mangifera indica* L.) fruit is an economic crop in over 100 countries and its global production is rising (*1*). It is a seasonal fruit and is a high-volume export of Thailand. Several well-known cultivars of mango are grown in this country. These include Nam Dok Mai, Chok Anan and Kaew. Chok Anan cultivar is well known in the mango processing industry (*2*). Mango is processed into products including puree, fruit leather, nectar, pickles and canned slices (*3*). Its peel is a major waste of industrial processing or fresh fruit. It comprises 7–24% of the total mass of mango fruit (*3*). Several researchers have found that mango peel is a major source of pectin, proteins and carbohydrates, and that it also contains phenolic compounds (mangiferin, quercetin, rhamnetin, ellagic acid, kaempferol, *etc*.), which are important antioxidants. These can be used as functional food or nutraceuticals (*4*). The presence of phenolic compounds in the diet may provide a defensive activity against some chronic degenerative diseases that entail oxidative stress (*5*).

Potato (*Solanum tuberosum*) is the fourth most significant food crop worldwide. It is used for both animal feed and human food production (*6*). It can be prepared in various ways. Potato handling, storage and processing can cause tissue damage and black and/or brown discoloration *via* an enzymatic browning reaction involving polyphenol oxidase (PPO) (*7*). Previous studies revealed that the phenolic compounds from rice bran extract can effectively inhibit enzymatic browning in potato (*8*–*10*).

PPO is the main enzyme that catalyzes the browning reaction in fresh-cut fruits (*11*). In the presence of oxygen, PPO catalyzes the oxidation of *o*-diphenols to *o*-quinone and the hydroxylation of monophenols to *o*-diphenol, which can undergo self-polymerization or interact with other compounds that subsequently form brown pigments (*12*). Enzymatic browning is a significant problem with fresh-cut fruits and vegetables such as apples, bananas, grapes, potatoes, lettuce and other leafy vegetables (*13*). Browning usually degrades the quality of fruit and vegetable due to colour changes which reduce their market value (*14*). Many methods are used to inhibit PPO activity. Generally, physical methods have been employed such as heating and ultrasound as well as the use of chemicals such as ascorbic and citric acids, and sulfites. Sulfites are a more effective browning inhibitor, but it has been reported that they may affect human health (*15*). Therefore, there is a consumer demand for the use natural PPO inhibitors to replace the synthetic compounds. Mango peel is a waste product from the fruit processing industry that is a rich source of antioxidative phenolic compounds (*3*, *4*). Therefore, the goal of the present work is to examine the inhibitory effects of mango peel extract using ultrasound-assisted extraction and its phenolic compounds as natural inhibitors of enzymatic browning in potato puree.

## MATERIALS AND METHODS

### Materials and chemicals

Potatoes (*Solanum tuberosum* L.) were bought from a local shop in Bangkok, Thailand. Mango (*Mangifera indica* L. cv. Chok Anan) peel was obtained from the Peace Canning (1958) Co. Ltd., Chiangmai, Thailand. We obtained Folin-Ciocalteu phenol reagent from Merck (Darmstadt, Germany). Triton X-100 and l-ascorbic acid were bought from Fluka (Steinheim, Germany). Sodium carbonate, polyvinylpyrrolidone, catechol, citric acid, sodium disulfite, gallic and protocatechuic acids used as a phenolic acid standard were obtained from Sigma-Aldrich, Merck, Steinheim, Germany. Mangiferin and ellagic acid were obtained from Sigma-Aldrich, Merck, Buchs, Switzerland. Acetic acid, and methanol were obtained from Merck (Darmstadt, Germany). Acetonitrile was obtained from Mallinckrodt (Phillipsburg, NJ, USA) and used in HPLC analyses.

### Preparation of mango peel liquid nitrogen powder

Mango peel was blended with liquid nitrogen in a blender (34B299; Waring, Stamford, CT, USA) until a mango peel liquid nitrogen powder was obtained using the same method as the one used to prepare pineapple shell powder (*16*). The powder was stored at –18 °C until use.

### Ultrasound-assisted extraction of phenolic  compounds from mango peel powder

The mango peel powder was mixed with distilled water in a ratio 1:6 (*m*/*V*). The first experiment was performed to select an ultrasound-assisted extraction time. Samples were treated at 25 °C for 15, 30 and 45 min in an ultrasonic bath (model DT 255H; Bandelin Electronic, Berlin, Germany) at a constant frequency of 50 kHz and power of 160 W. The most effective ultrasound-assisted extraction time from prior studies was selected. Next, the extraction by ultrasound combined with stirring for 15, 30 and 45 min at room temperature was further investigated. The homogenate was centrifuged (Sorvall RC–5C Plus; Thermo Fisher Scientific, Willmington, DE, USA) at 10 000×*g* and 25 °C for 30 min. Then the total phenolic content of samples was evaluated as described below. Concentrations of all tested mango peel extracts were expressed as equivalent mass (in g, on dry mass basis) of mango peel per mL of solvent (distilled water).

### Determination of total phenolic content

Total phenolic content in mango peel extract was determined using the Folin-Ciocalteu method (*17*). Briefly, 50 µL of the diluted sample (or standard gallic acid solution) were added to 1950 µL of distilled water and 250 µL of Folin-Ciocalteu reagent solution, and then 750 µL of 7% sodium carbonate were added after 6 min. After incubation at room temperature for 2 h in the dark, the absorbance of the reaction was measured at 765 nm by spectrophotometer GENESYS 10S (Thermo Fisher Scientific, Waltham, MA, USA). Gallic acid was used as a standard, and the results were expressed in milligrams of gallic acid equivalents (GAE) per 100 g of mango peel (dry mass basis).

### Browning inhibition

The potatoes were peeled and then blended with mango peel extract or commercial inhibitors (citric acid, ascorbic acid, or sodium disulfite at the concentration of 20 mg/L) at ratio 2:1 (*m*/*V*) for 20 s. Sample colour values were measured using a colorimeter (CM-3500D; Konica Minolta, Ramsey, NJ, USA) after 0, 1, 2, 3, 4, 5 and 6 h of storage at room tempera- ture. The browning value of samples at each storage time was calculated using the following equation:

Browning value=(ΔL*/*L*_0_*)·100 /1/

where ΔL* is equal to *L*_0_*– L*, L* is the lightness value at a selected time and *L*_0_* is the initial lightness (*18*). Similarly, browning inhibition (in %) was calculated as follows (*19*):

Browning inhibition=(Browning value_control_–Browning value _inhibitor_)·100/Browning value_control_ /2/

### Polyphenol oxidase inhibition

#### PPO preparation

The preparation of PPO from potato flesh was modified from the method of Galeazzi *et al*. (*20*) as follows: potato flesh (100 g) was blended in a blender (34B299; Waring) for 20 s with 100 mL of cold 0.1 M sodium phosphate buffer (pH=6.6) containing 0.5% Triton X-100 and 1% polyvinylpyrrolidone. The homogenate was then centrifuged (Sorvall RC-5C Plus; Wilmington, DE, USA) at 15 000×*g* at 4 °C for 30 min. The supernatant was filtered through a regular cheese cloth, then the extract was collected at 4 °C and referred to as a crude enzyme.

#### PPO activity assay

PPO activity was estimated by the modified method of Lee (*21*) as follows. Briefly, 1.0 mL of 0.2 M catechol (as the substrate) in 0.05 M sodium phosphate buffer, pH=6.6, was mixed with 1 mL of distilled water and 0.9 mL of 0.05 M sodium phosphate buffer, pH=6.6, then incubated for 30 s. After that, 0.1 mL of crude enzyme was added and gently mixed. Its absorbance was rapidly measured every 10 s for 1 min at 420 nm using a spectrophotometer (GENESYS 10S; Thermo Fisher Scientific). One unit of PPO activity was defined as the amount of enzyme that caused an increase in the absorbance at 420 nm by 0.001/min at 25 °C, pH=6.6.

#### Evaluation of mango peel extract and various antibrowning agents on PPO inhibition

The effect of mango peel extract (as an equivalent to 0.04 g/mL mango peel on dry mass basis) and antibrowning agents (citric and ascorbic acids including sodium disulfite) at a final concentration of 20 mg/L on potato PPO activity was tested with the same method of PPO activity determination by adding each inhibitor (1 mL) instead of distilled water. The inhibition (in %) was calculated as described by Chaisakdanugull *et al*. (*22*):

Inhibition=[(activity of control–activity of inhibitor)/activity of control]∙100 /3/

### HPLC-DAD analyses of phenolic compounds

Phenolic compounds were separated using Waters HPLC (Waters, Milford, Ireland) equipped with Empower^TM^ 2 software, a model 600 controller gradient pump, a Waters model 2707 autosampler and a Waters model 2998 diode array detector. The HPLC column was Hypersil ODS (4.6 mm×250 mm i.d., 5 μm; Agilent, Amsterdam, the Netherlands), with a 4.0 mm×4 mm i.d., 5 μm Hypersil ODS guard column (Agilent, Darmstadt, Germany). The mobile phase was 1% (by volume) acetic acid in methanol (eluent A) and 1% (by volume) acetic acid in water (eluent B). The following solvent gradients were applied: 10–35% A (10 min), 35–42% A (15 min), 42–75% A (10 min), 75-75% A (5 min) and 75 to 10% A (10 min). The injection volume used for all samples was 10 μL at a flow rate of 1 mL/min. The UV/Vis detection was recorded at 280 nm for gallic, protocatechuic and ellagic acids and at 370 nm for mangiferin (modified from Li *et al*. (*23*)).

### The inhibitory effect of phenolic compounds from mango peel extract on PPO

To confirm the effect of the major phenolic compounds found in mango peel extract on PPO activity inhibition, ellagic, gallic and protocatechuic acids and mangiferin were prepared at the same concentration as the mango peel extract from the HPLC analysis. The effects of these phenolic compounds were assayed for potato polyphenol oxidase inhibition as described above.

### Inhibition kinetics of mango peel extract on potato PPO

The inhibition kinetics of mango peel extract on potato PPO was determined according to the modified method of Kubglomsong and Theerakulkait (*19*) by monitoring the PPO activity at substrate (catechol) concentrations of 0.025–0.2 M. Data were plotted as 1/*v* against 1/[S] (Lineweaver-Burk plot). To specify the type of mango peel extract inhibition on potato PPO, four mango peel extract concentrations: 0, 0.01, 0.02 and 0.04 g/mL were tested using 0.2 M catechol as substrate, again using Lineweaver-Burk plots.

### Statistical analyses

Analyses were all done in triplicate. Statistical significance was assessed using a univariate analysis of variance with SPSS v. 17 (*24*). Significant differences (p<0.05) among treatments were detected using Duncan’s multiple range test (*19*).

## RESULTS AND DISCUSSION

### Ultrasound-assisted extraction of phenolic compounds from mango peel extract

Mango peel extract was extracted by blending with liquid nitrogen and using ultrasound-assisted extraction for 15, 30 and 45 min. The total phenolic compound content as GAE on dry mango peel mass basis was the highest (972 mg/100 g) when using a 15-minute ultrasonic treatment. It was more productive than treatments for 30 and 45 min. When using extraction times of 15 and 30 min, the total phenolic content (972 and 939 mg/100 g) was not significantly different, but it was higher than with extraction time of 45 min (p<0.05) (885 mg/100 g). This might be due to the destruction of phenolic compounds by ultrasound with increasing extraction time. The mango peel extract blended with liquid nitrogen as a freezing agent was useful for controlling enzymatic browning of the mango peel. The ultrasonic processing was vigorous enough to cause damage to cells and increase the release of cell content, leading to increased total phenolic yield. Altemimi *et al*. (*25*) reported that exposure to ultrasound could increase the total phenolic compound content in peach and pumpkin extracts. Ultrasound also effectively extracted phenolic compounds from orange (*26*) and pomegranate peels (*27*).

The total phenolic content of mango peel extract obtained by ultrasound for 15 min in combination with stirring for 15 min was the highest compared to the extraction with ultrasound for 15 min combined with stirring for 30 and 45 min, and ultrasound only for 15 min (control). The mass fractions of mango peel extract phenolics obtained by ultrasound for 15 min in combination with stirring for 15, 30 and 45 min were 972, 939 and 885 mg/100 g, respectively (p<0.05; data not shown), while their mass fraction in the control (extracted by ultrasound only) was 880 mg/100 g. This is similar to the extraction by ultrasound for 15 min combined with stirring for 45 min (p>0.05). The concentration of phenolic compounds decreased with increasing time of stirring and extraction. This might be due to the breakdown of active phenolic compounds (*28*).

### Effects of mango peel extract and various antibrowning agents on potato puree browning

**Fig. 1:**
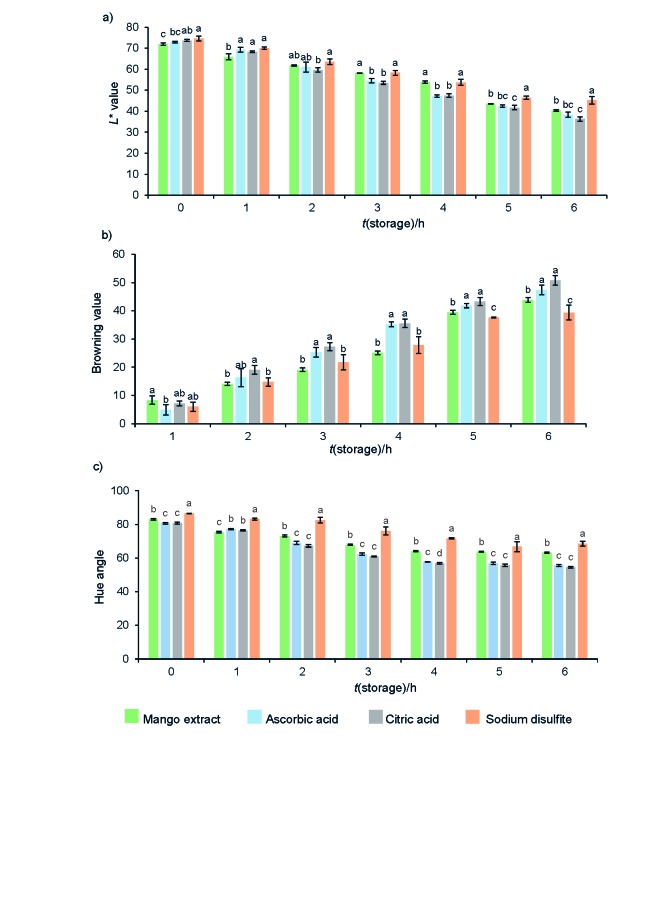
Results for: a) L* values, b) browning values ((∆L*/*L*_0_*)·100) and c) hue angle of potato puree blended with mango peel liquid nitrogen powder extract (*γ*=0.04 g/mL) and various antibrowning agents (ascorbic acid, citric acid and sodium disulfite) at *γ*=20 mg/L and storage time of 0–6 h at 25 °C. Mean values with different letters are significantly different (p<0.05) for the same storage time

From our previous research, we found that mango peel extract inhibited enzymatic browning of potato puree to a higher degree than of banana puree (*29*). Therefore, potato was chosen for further study. The L* results of potato puree treated with mango peel extract were compared with those when a commercial antibrowning agent was added (citric acid, ascorbic acid, or sodium disulfite) by testing them at a final concentration of 20 mg/L (Fig. 1a). The L* values of potato puree treated with mango peel extract were higher than those of treated with 20 mg/L of citric acid and stored for 3–6 h (p<0.05). The L* values of potato puree treated with mango peel extract were higher than those when treated with 20 mg/L ascorbic acid and stored for 3 and 4 h (p<0.05). Additionally, L* values of puree samples treated with 20 mg/L sodium disulfite were higher than those when treated with ascorbic or citric acid (final concentration of 20 mg/L) and stored for 5 and 6 h (p<0.05). However, after storage for 2 to 4 h, L* values of potato puree treated with sodium disulfite were slightly higher than of the puree treated with mango peel extract (p>0.05). After 6 h of storage, L* values of the puree treated with mango peel extract, ascorbic acid, citric acid and sodium disulfite were 45, 40, 38 and 36, respectively, showing that mango peel extract was more effective in potato puree browning inhibition than ascorbic or citric acid.

Fig. 1b shows the browning values of potato puree mixed with various antibrowning agents after storage at room temperature for up to 6 h. Values for potato puree treated with mango peel extract were lower than of samples treated with ascorbic or citric acid (final concentration 20 mg/L) at storage times of 3 to 6 h (p<0.05). Mango peel extract treated potato puree exhibited slightly lower browning values, roughly similar to those after treatment with 20 mg/L ascorbic acid after storage for 2 h (p>0.05). Moreover, samples treated with ascorbic acid had slightly lower browning values than samples treated with citric acid after storage for 3–6 h. Potato puree treated with 20 mg/L sodium disulfite had the lowest browning values after storage for 5 and 6 h. Browning values of potato puree treated with sodium disulfite, mango peel extract, ascorbic and citric acid after 6 h of storage were 39, 44, 47 and 51, respectively. The results indicate that mango peel extract could decrease browning of potato puree during storage more than other commercial antibrowning agents.

Nicoli *et al*. (*30*) reported that a decrease in the hue angle indicated the changes of colour from yellow to orange and a decrease in L* value also indicated darkening of the colour. Fig. 1c shows that hue angles of potato puree treated with mango peel extract were larger than those when treated with ascorbic or citric acid (final concentration 20 mg/L) after storage for 2–6 h (p<0.05). Potato puree treated with 20 mg/L ascorbic acid had slightly higher hue angle value, similar to that from the treatment with 20 mg/L citric acid after storage for 1, 2, 3, 5 and 6 h. Additionally, hue angles for potato puree treated with 20 mg/L of sodium disulfite were larger than those when treated with mango peel extract, ascorbic or citric acid after all storage times (69, 63, 56 and 55, respectively; p<0.05). From the browning values, L* values and hue angles, it can be concluded that mango peel extract had higher antibrowning effect on potato puree than citric or ascorbic acid, but lower than sodium disulfite. Sukhonthara and Theerakulkait (*31*) reported that rice bran extract inhibited enzymatic browning of potato puree more than the samples treated with ascorbic or citric acid (final concentration 20 mg/L). However, sodium disulfite was determined to be the most effective inhibitor of potato PPO.

### Effects of mango peel extract and various antibrowning agents on potato polyphenol oxidase

The potato PPO inhibition by mango peel extract (0.04 g/mL) was much higher than by other antibrowning agents (citric and ascorbic acids, and sodium disulfite at a final concentration of 20 mg/L), with inhibition equal to 62%, compared to 10 and 6% for citric and ascorbic acid, respectively. Sodium disulfite showed the highest potato PPO inhibition. Sukhonthara and Theerakulkait (*31*) reported that rice bran extract combined with either ascorbic or citric acid increased the inhibition of enzymatic browning in potato. Duangmal and Owusu Apenten (*32*) found that sodium disulfite had the highest inhibitory effect on potato PPO, because sulfite reacts with the disulfide bonds in PPO and leads to inactivation and change of its tertiary structure.

### Kinetic study

**Fig. 2:**
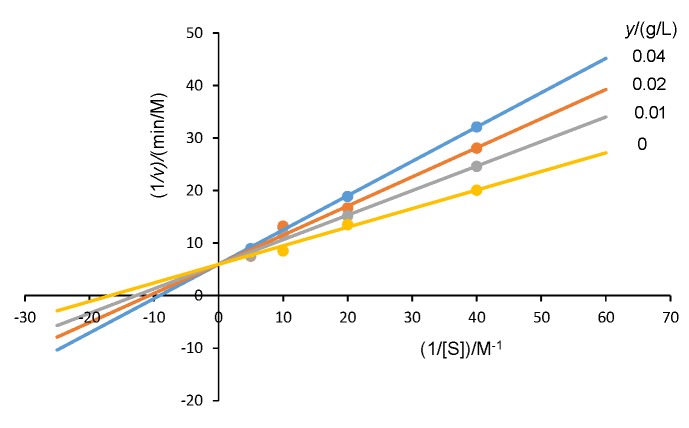
Lineweaver-Burk plots (*v*=reaction rate (M/min) and [S]=equilibrium concentration of substrate (M)) for the inhibition of potato polyphenol oxidase by mango peel liquid nitrogen powder extract

The inhibition kinetics of mango peel extract on potato PPO was analysed with Lineweaver-Burk plots as shown in Fig. 2. The results show that mango peel extract exhibited a competitive inhibitory effect on potato PPO by using catechol as a substrate. Maisuthisakul and Gordon (*2*) reported that mango seed kernel extract was also a competitive inhibitor, confirmed by its constant *v*_max_ value. The structure of most competitive inhibitors is similar to that of the substrate. Nevertheless, Lou *et al*. (*33*) found that the compound 3′,5′-di-C-β-glucopyranosylphloretin from calamondin peel is competitive inhibitor since the B ring of the 4-hydroxy group closely resembles the structure of the substrate. 3-Hydroxyphloretin isolated from Formosan apple acted as a competitive inhibitor and exhibited reduced tyrosinase activity (*34*).

The inhibition percentage was plotted against the concentration of the inhibitor to determine the amount of mango peel extract needed for 50% inhibition (IC_50_) of potato PPO activity. In this research, the IC_50_ value was estimated to 0.3 mg/mL from the plots of concentration *versus* the inhibition percentage. Other researchers also reported the IC_50_ values of longan fruit extract (2.9–3.2 mg/mL) (*35*), and immature calamondin peel extract (0.87 mg/mL) (*33*) for PPO activity inhibition. Therefore, this result indicated that mango peel extract has a strong inhibitory activity that could inhibit the PPO activity at a low concentration.

### Effects of various phenolic acids from mango peel extract on potato PPO inhibition determined by HPLC

**Fig. 3:**
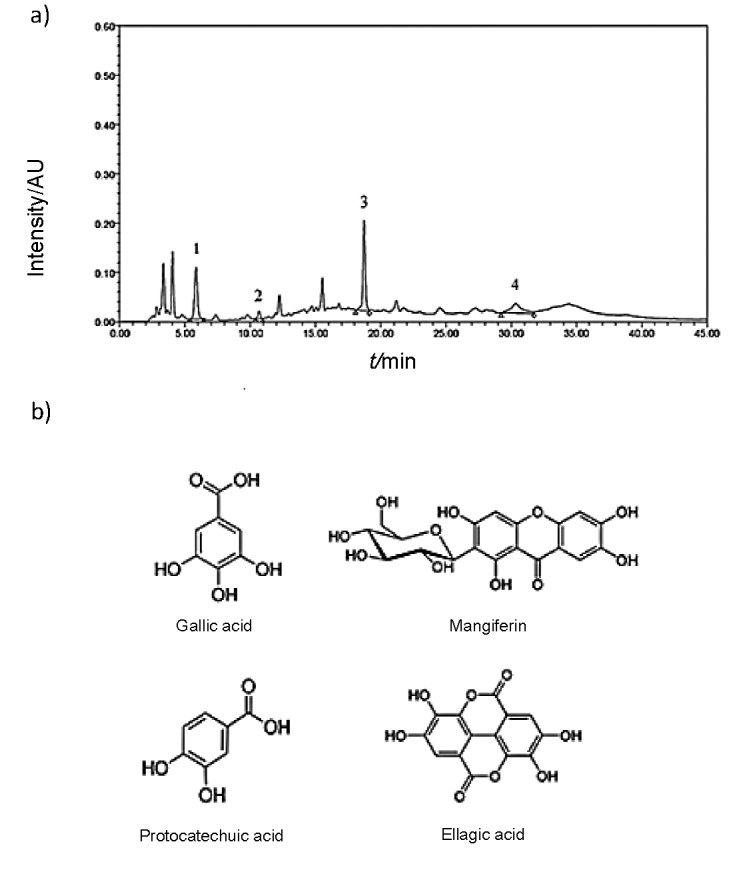
Chromatogram of phenolic compounds from: a) mango peel liquid nitrogen powder extract identified by HPLC. 1=gallic acid, 2=protocatechuic acid, 3=mangiferin and 4=ellagic acid, and b) chemical structure of phenolic compounds in the mango peel extract

The phenolic compounds in mango peel extract (including ellagic, gallic and protocatechuic acids and mangiferin) were measured using HPLC analysis. A standard chromatogram with the chemical structure of these compounds is shown in Fig. 3. Table 1 shows that the mango peel extract had the highest concentration of mangiferin, followed by ellagic, gallic and protocatechuic acids (220, 105, 71 and 13 μg/mL, respectively). Ajila *et al*. (*36*) found that the phenolic compounds found in the peel of Badami variety of mango were quercetin, mangiferin, syringic and ellagic acids, and pentoside. The peel of Raspuri mango variety contained ferulic, gallic, protocatechuic and syringic acids, rutin, quercetin and kaempferol (*37*). The role of phenolic compounds as possible inhibitors of PPO activity was experimentally examined using model systems in which each of the phenolic compounds was prepared at the same concentration as the mango peel extract for HPLC analysis, as shown in Table 1. The relative inhibitory activities on potato PPO were in the following order: mangiferin=gallic acid=protocatechuic acid>ellagic acid (p<0.05). Mangiferin exhibited slightly higher potato PPO inhibition than gallic and protocatechuic acids. Ellagic acid had the lowest inhibitory effect on potato PPO (p<0.05). Mangiferin and gallic and protocatechuic acids have two hydroxyl groups in the structure (Fig. 3b). This structure is an analogue of the diphenolase substrate, catechol. It may inhibit the activity of PPO through a competitive inhibition. Nagendra Prasad *et al*. (*38*) reported that the inhibitory activity of an enzyme may depend on the hydroxyl group of the phenolic compound in litchi seed extracts. These groups were able to form hydrogen bonds with the active site of the enzyme, leading to decreased enzymatic activity. The hydroxyl group of some inhibitors can bind to the active site of an enzyme resulting in steric hindrance or a change in the structure of the enzyme. Mangiferin and gallic and protocatechuic acids also have a conjugated structure made up of a benzene ring or a carboxyl group. Thus, this structure may contribute steric hindrance to the enzyme active site. Qiu *et al*. (*39*) reported that 4-hydroxycinnamic acid could inhibit diphenolase activity since it is a large conjugated compound consisting of a benzene ring, an ethylenic linkage and a carboxyl group that provides steric hindrance to the enzyme active site. Protocatechuic acid from black rice bran extract also had inhibitory effect on enzymatic browning (*40*). Macrae and Duggleby (*41*) reported that the *p*-coumaric, ferulic and cinnamic acids exhibited the most potent inhibitory effect against potato PPO. These acids from full-fatted and commercially defatted rice bran extracts exhibited inhibitory effects against enzymatic browning on potato and apple PPO (*10*).

**Table 1:** Concentrations of phenolic compounds separated by HPLC from mango peel liquid nitrogen powder extract and their inhibition of potato polyphenol oxidase activity

Mango peel extract	Gallic acid	Protocatechuic acid	Mangiferin	Ellagic acid
*γ*/(µg/mL)	(70.5±8.9)^c^	(13.1±2.9)^d^	(220.2±8.5)^a^	(104.9±11.6)^b^
Inhibition/%	(77.1±2.0)^a^	(77.1±2.0)^a^	(80.2±0.5)^a^	(13.9±4.0)^b^

Mean values with different letters in the same row are significantly different (p<0.05)

## CONCLUSIONS

Extraction of phenolic compounds from mango peel liquid nitrogen powder with ultrasonication for 15 min combined with stirring for 15 min showed a high total phenolic compound content and strong potato polyphenol oxidase (PPO) inhibitory effect. The extract was effective in inhibiting browning in potato puree during storage for 1-6 h. Our kinetic inhibition study of potato PPO by mango peel liquid nitrogen powder extract revealed competitive inhibition. The major phenolic compounds present in mango peel extract were mangiferin and gallic, protocatechuic and ellagic acids, as determined by HPLC. Mangiferin and gallic and protocatechuic acids had higher inhibitory effects on potato PPO activity than ellagic acid at the concentration present in the extract. This indicated that mangiferin and gallic and protocatechuic acids play an important role in potato PPO inhibition. Therefore, mango peel extract has a potential for use as a natural antibrowning agent in potato puree.
